# Hepatocytes maintain greater fluorescent bile acid accumulation and greater sensitivity to drug‐induced cell death in three‐dimensional matrix culture

**DOI:** 10.14814/phy2.12198

**Published:** 2014-12-18

**Authors:** John W. Murray, Dennis Han, Allan W. Wolkoff

**Affiliations:** 1 Department of Anatomy and Structural Biology, Division of Gastroenterology and Liver Diseases, Marion Bessin Liver Research Center, Albert Einstein College of Medicine, Montefiore Medical Center, Bronx, New York

**Keywords:** Collagen sandwich, fluorescent bile acid (FBA), hepatocyte culture

## Abstract

Primary hepatocytes undergo phenotypic dedifferentiation upon isolation from liver that typically includes down regulation of uptake transporters and up regulation of efflux transporters. Culturing cells between layers of collagen in a three‐dimensional (3D) “sandwich” is reported to restore hepatic phenotype. This report examines how 3D culturing affects accumulation of fluorophores, the cytotoxic response to bile acids and drugs, and whether cell to cell differences in fluorescent anion accumulation correlate with differences in cytotoxicity. Hepatocytes were found to accumulate fluorescent bile acid (FBA) at significantly higher levels than the related fluorophores, carboxyfluorescein diacetate, (4.4‐fold), carboxyfluorescein succinimidyl ester (4.8‐fold), and fluorescein (30‐fold). In 2D culture, FBA accumulation decreased to background levels by 32 h, Hoechst nuclear accumulation strongly decreased, and nuclear diameter increased, indicative of an efflux phenotype. In 3D culture, FBA accumulation was maintained through 168 h but at 1/3 the original intensity. Cell to cell differences in accumulated FBA did not correlate with levels of liver zonal markers L‐FBAP (zone 1) or glutamine synthetase (zone 3). Cytotoxic response to hydrophobic bile acids, acetaminophen, and phalloidin was maintained in 3D culture, and cells with higher FBA accumulation showed 12–18% higher toxicity than the total population toward hydrophobic bile acids (*P* < 0.05). Long‐term imaging showed oscillations in the accumulation of FBA over periods of hours. Overall, the studies suggest that high accumulation of FBA can indicate the sensitivity of cultured hepatocytes to hydrophobic bile acids and other toxins.

## Abstract (precis)

These studies use automated image analysis and fluorescent dye accumulation to demonstrate that 3D culturing enhances organic anion accumulation and cytotoxic response in long‐term hepatocyte cultures. The level of anion accumulation was found to vary through days in culture and also between single cells, and higher fluorescent bile acid accumulation correlated with higher toxic response to hydrophobic bile acids.

## Introduction

Primary hepatocytes undergo phenotypic change, or dedifferentiation, upon isolation from liver and subsequent laboratory culturing. This dedifferentiation response, which can vary depending on culture conditions and animal species, involves an inflammatory response arising from ischemia, tissue disruption, and partial digestion of plasma membrane protein that occurs during the isolation procedure (Fraczek et al. 2013; Godoy et al. 2013). Typical features of hepatocyte dedifferentiation include cell spreading, loss of polarity, sporadic cell death but diminished apoptotic/necrotic response to stimuli, and major changes in protein expression including loss of liver specific CYP P450 enzymes (Bissell and Guzelian 1980; Fraczek et al. 2013; Godoy et al. 2013). One characteristic of hepatocytes is their ability to take up and secrete organic anions, particularly bile acids, and organic anion transport mechanisms are also perturbed during primary culture. For instance, in rat hepatocytes the uptake transporters, ntcp, and oatp1a1 were shown to decrease by approximately 50% within 24 h of primary cell culture while expression of excretory transporters Mrp2 and BSEP were maintained or increased (Liang et al. 1993; Rippin et al. 2001). The three‐dimensional extracellular matrix “sandwich” culture technique (3D culturing) is reported to maintain the hepatocyte phenotype for weeks of culture (Dunn et al. 1989; LeCluyse et al. 1994; Swift et al. 2010; Treyer and Musch 2013) and the following studies examine in detail how organic anion transport changes during primary culture.

High‐content cell imaging represents a means to measure phenotypic variability in individual cells, and this report also investigates the use of the fluorescent bile acid (FBA), chenodeoxycholylglycylamidofluorescein (CDCGamF, that is, chenodeoxycholate coupled to fluorescein) as a marker of hepatocyte phenotype. CDCGamF and other FBAs are substrates for bile acid transporters (Mita et al. 2006; Murray et al. 2011) and accumulate to various degrees in hepatocytes in culture. As compared to native bile acids, transport and biotransformation of FBA is generally lower, whereas cellular retention is high. The fluorescein group itself is sensitive to pH and adds additional negative charge to the bile acid. None‐the‐less, image‐based anion transport assays provide a means to quantify the inherently variable nature of bile acid transport in primary cell culture in high detail. These studies probed to what extent fluorescent anion transport is maintained in 3D versus 2D rat hepatocyte culture, whether the toxicity response to bile acids is maintained in 3D culture, and finally whether high accumulation of fluorescent bile acid on a per‐cell basis corresponds with the toxic response to native bile acids.

## Methods

### Reagents

Reagents were from Sigma‐Aldrich (St. Louis, MO) unless noted. Fluorescent bile acid, CDCGamF (here alternately referred to as FBA) (Mita et al. 2006), was obtained from Dr. Alan Hofmann (University of San Diego, CA), structure was confirmed by mass spectrometry. Coverslip‐bottomed chambers were from MatTek (Ashland, MA), and In Vitro Scientific (Sunnyvale, CA). 96‐well plates were from BD Biosciences (Cat # 353872). Antibodies to L‐FABP (ab7847) and glutamine synthetase (Ab64613) were from Abcam (Cambridge, MA). All animal procedures were approved by the University Animal Use Committee. Male Sprague–Dawley rat hepatocytes were isolated by two‐step collagenase perfusion (Neufeld 1997), temporarily stored in L‐15 medium, and viable cells were additionally purified by sedimentation in 35% isotonic Percoll. Cells were cultured in Williams E media lacking phenol red and supplemented with 0.1 *μ*mol/L dexamethasone, 2 mmol/L Glutamax, 100 U/mL penicillin‐streptomycin, and 1× ITS (10 *μ*g/mL insulin, 5.5 *μ*g/mL transferrin, 6.7 ng/mL sodium selenite (Life Technologies, Thermo Fisher Scientific, Waltham, MA). Cells were plated at 1200 cells/mm^2^ on coverslip‐bottomed chambers or 96‐well plates that had been coated by 5 min exposure to 0.1 mg/mL rat tail collagen (Cat # A1048301, Life Technologies). To create 3D cultures, medium including 0.15 mg/mL rat tail collagen on day 0, and 0.06 mg/mL thereafter was allowed to gel. This minimal concentration of collagen and promoted hepatocyte phenotype, but could be removed without damaging the cells. The collagen overlay was found to delay the cellular accumulation of fluorescent dyes and was therefore removed during the wash procedures, prior to application of the dyes, for all applicable experiments. The medium was changed on day 0, day 1, and every 2 days thereafter (but not immediately prior to experiments). Washes and incubations were in serum‐free medium (SFM, 135 mmol/L NaCl, 1.2 mmol/L MgCl_2_, 0.8 mmol/L MgSO_4_, 28 mmol/L dextrose, 2.5 mmol/L CaCl_2_, and 25 mmol/L Hepes, pH 7.4) unless otherwise noted.

### Fluorescent anion accumulation versus time in culture

**Figure 1.**
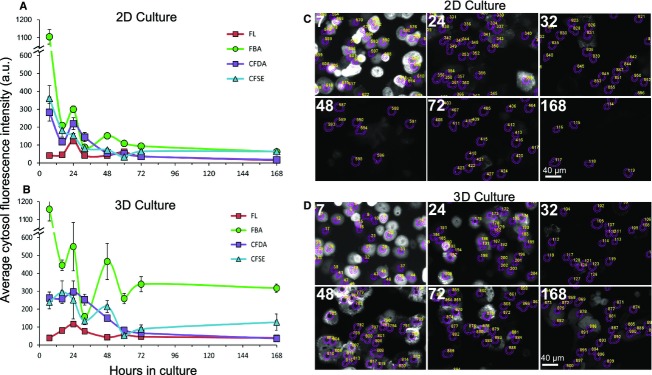
Fluorescent bile acid accumulation is maintained in 3D culture. Primary rat hepatocytes were assayed for their ability to accumulate a series of fluorescence anions, FL (fluorescein), FBA (fluorescent bile acid, i.e., CDCGamF), CFDA (carboxyfluorescein diacetate), CFSE (carboxyfluroescein succinimidyl ester) at 1 umol/L during long‐term culture under 2D (A, C) or 3D (B, D) extracellular matrix conditions. Experiments were scored by automated analysis and the average cytosol intensity is shown in arbitrary units (a.u., i.e., the camera pixel intensity subtracted from background). Representative images of FBA accumulation are shown in (C) and (D) along with the cytosolic region of interest used for quantification, with hours in culture indicated. Error bars are standard error of the mean of three experiments.

**Figure 2.**
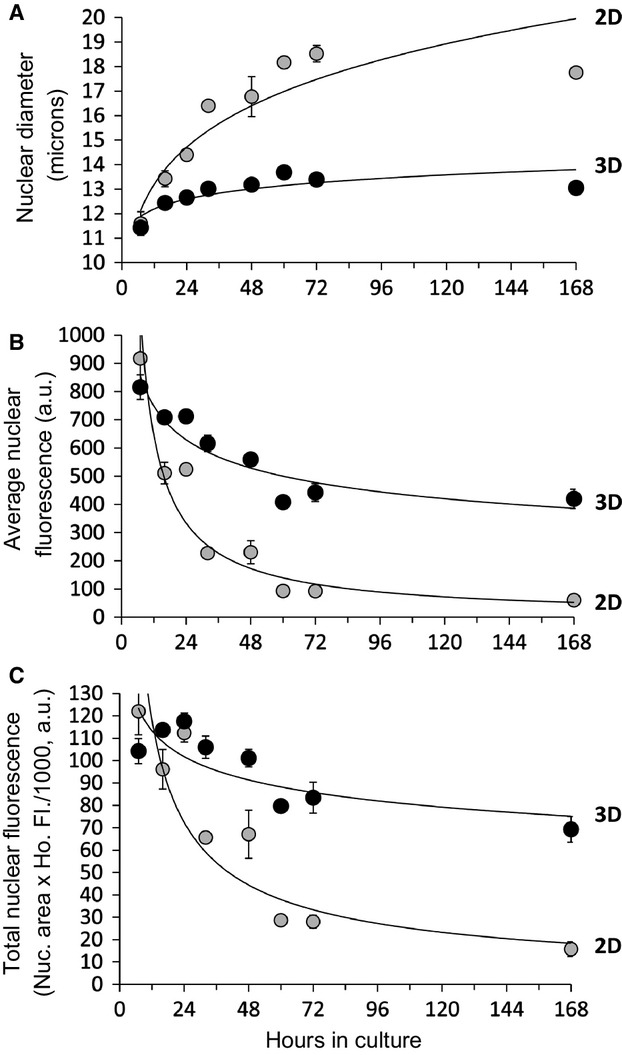
Nuclear diameter increases and accumulation of Hoechst nuclear stain decreases for primary hepatocytes under 2D culture. Hepatocytes of Fig. 1 were analyzed for their average nuclear diameter (A) and nuclear fluorescence intensity from Hoechst dye (B). The total nuclear fluorescence (C) (i.e., the integrated Hoechst fluorescent intensity) decreased over time, indicating that Hoechst dye was being excluded from nuclei at later time points. Lines indicate curve fit of the data using the power function (*y* = *ax*^b^). For simplicity, data from the control (i.e., lacking fluorescent anion) are shown; results from the other experiments were similar. Error bars are standard error of the mean of three experiments.

Cells were cultured on 96‐well plates for up to 168 h (7 days) (Figs. 1, 2). At various times, the top layer of collagen (if present) was aspirated and wells were washed twice, followed by 15 min incubation with 1 *μ*mol/L fluorescent anion, followed by 10 min incubation with 20 *μ*mol/L Hoechst 33342 and 20 nmol/L Lysotracker Red DND‐99, followed by washing five times and imaging, all at 37°C. Experiments were performed in triplicate with at least eight random fields captured per experiment. Microscope fluorescence intensity was calibrated for each set of readings.

### Immunofluorescence correlation

Experiments were performed similarly to those described in Murray et al. (2011) (Fig. 4). Rat hepatocytes were plated on Matrigel (Corning Inc, Corning, NY) coated coverslips for 4 h, then incubated with 250 nmol/L CDCGamF in SFM for 10 min at 37°C, and imaged with recorded stage positions. Cells were washed in PBS and fixed in 4% paraformaldehyde containing 10 mmol/L Hepes, pH 7.4, for 10 min, followed by permeabilization in 0.1% Triton X‐100 in PBS for 5 min. Anti LFABP and anti glutamine synthetase antibodies were incubated at 1:50 dilution for 1 h followed by overnight incubation in appropriate secondary antibody (containing Cy5 or Cy3 fluorophores). Unbound antibody was washed and cells stained with 1 *μ*g/mL Hoechst and the exact field positions were re‐imaged. Images were scored automatically by identifying individual cells via Hoechst fluorescence, segmenting the nuclei, and measuring cell fluorescence within a cellular ROI, consisting of the nuclear border to 3 microns beyond the nuclear border.

### Perfusion of liver with FBA

Portal veins were canulated as for hepatocyte isolation followed by perfusion for 2 min with Krebs‐Ringer buffer (120 mmol/L NaCl, 24 mmol/L NaHCO_3_, 20 mmol/L Glucose, 10 mmol/L Hepes, 4.8 mmol/L KCl, 1.2 mmol/L MgSO_4_, 1.2 mmol/L KH_2_PO_4_, 1.2 mmol/L CaCl_2_) followed by 400 *μ*L of 125 *μ*mol/L CDCGamF, and then 300 *μ*L of 1 mmol/L Hoechst (Fig. 5). This was followed by an additional 15 min of perfusion. Liver segments were then snap frozen in 2‐methylbutane cooled in dry ice and subsequently sliced, affixed to slides, fixed in 100% methanol, and imaged. Methanol was found to retain FBA better than paraformaldehyde or ethanol.

### Cell death as a function of high medium and low FBA accumulation

Hepatocytes were plated on collagen‐coated dishes in cell culture media supplemented with 100 mmol/L Hepes and diluted with H_2_O to maintain osmotic pressure (Fig. 6). 100 nmol/L CDCGamF, 10 *μ*mol/L propidium iodide, and 1 *μ*mol/L Hoechst were added for 5 min, multiple fields of cells were then imaged, followed by addition of either 500 *μ*mol/L taurolithocholic acid, 150 *μ*mol/L glycochenodeoxycholic acid, 10 mmol/L acetaminophen, or equivalent solvent (0.5% DMSO: ethanol mixture). The fields were then re‐imaged once every 10 min for 30 h at 37°C to observe cell death. Individual cells were identified by Hoechst nuclear stain, and FBA and propidium iodide intensity were measured in the cellular ROI. Cell death within the first 30 h was measured by an increase in propidium iodide fluorescence. FBA fluorescence was measured in frame 1, it decreased with addition of the bile acids.

### Image analysis

**Figure 3.**
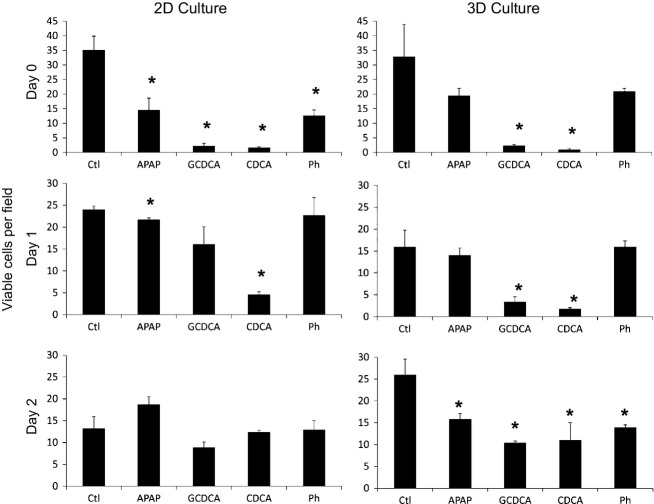
3D culturing maintains the cytotoxic response of primary hepatocytes to acetaminophen, hydrophobic bile acids, and phallodin. Rat hepatoctyes were cultured in 96‐well plates in 2D or 3D configuration and, after the indicated days in culture (Day 0, 1, 2), cells were exposed to either 150 *μ*mol/L glycochenodeoxycholic acid (GCDCA)), 150 *μ*mol/L chenodeoxycholic acid (CDCA), 10 mmol/L acetaminophen (APAP), or 50 *μ*mol/L phalloidin (Ph) for 14 h, followed by addition of 20 *μ*mol/L Hoechst and 10 *μ*mol/L propidium iodide for at least 10 min, followed by imaging. The *Y* axis indicates the number of viable cells per field. Each condition was performed in triplicate and eight random fields were acquired per experiment. Viable cells were scored by computer algorithm. Error bars are standard error of the mean, *P < 0.05, Student's t‐test compared to control.

Images were quantified using ImageJ (ImageJ, National Institutes of Health, Bethesda, MD, http://rsb.info.nih). For Fig. 1, a macro was created that segments (digitally selects) each nucleus as a region of interest (ROI) using Hoechst fluorescence and creates a cellular ROI 3 microns beyond the nuclear border. It applies these ROIs to the fluorescence channels for measurements. Image processing for nuclear segmentation included the spot enhancing filter (Daniel Sage, Biomedical Imaging Group, (Sage et al. 2005)) and automated thresholding using the triangle method. Damaged cells and debris were identified and excluded by their outlier status. Potential cells were considered outliers if their pixel intensity standard deviation was as follows: >mean ×15 or <mean ÷15 for anion fluorescence, >mean ×10 or <mean ÷5 for Lysotracker fluorescence (as vital dye), and >mean ×2.5 or <mean ÷5 for Hoechst fluorescence, >mean ×1.5 for Lysotracker intensity in nuclear ROI, <47 or >360 microns^2^ for nuclear area, or if circularity was <0.6. Nuclear diameter was the maximum Feret diameter. Anion fluorescent intensities were subtracted from control (vehicle, lacking fluorescent anion). For Hoechst, Lysotracker, and propidium iodide, background intensity (image mode) was subtracted. For Fig. 3, image processing and nuclei selection were performed similarly. Viable cells were scored as the number of qualifying nuclei (i.e., area >36 and <468 um^2^) with propidium iodide pixel intensity <200 in nuclear ROI, and Hoechst standard deviation <mean ×2 and >mean ÷2, and circularity exclusion as above. Image processing for Fig. 6 was similar except that smoothing and the convolve filter was used instead of spot enhancing for nuclear segmentation, and outliers included nuclear area <36 and >252 microns^2^ and circularity <0.05. More strenuous outlier removal was not needed for day 0 cells. Cells were determined to be nonviable in the first 30 h if propidium iodide cell fluorescence exceeded 100 units above background. Cell fluorescence was expressed as a ratio to Hoechst fluorescence.

### Imaging

Either confocal or epifluorescence microscopy was run with Metamorph Software (Molecular Devices LLC, Sunnyvale, CA) on an Olympus iX71 with an automated X‐Y‐Z stage (Applied Scientific Instrumentation, Eugene, OR) and 60× 1.4 NA oil, 20×, 0.75 NA, or 20× or 10× long‐working distance lenses. Epifluorescence imaging employed a DG‐4 xenon lamp (Sutter Instrument Co., Navoto, CA) with Dapi, Cy2, Cy3, Cy5 fluorescence and bright‐field channels and a cooled CCD camera (CoolSnap HQ, Photometrics, Tucson, AZ). Spinning disk confocal utilized PhotoFluor metal halide white light excitation (Chroma Technologies, Bellows Falls, VT) with similar channel capture using a CARV II spinning‐disk unit (Crisel Instruments, Rome, Italy) and an iXon 897 EMCCD camera (Andor Technologies, Belfast, Ireland).

## Results

### Fluorescent bile acid accumulation is maintained with 3D culturing, but at reduced levels compared to freshly isolated hepatocytes

Typically, primary hepatocytes will dedifferentiate from their absorptive, secretory epithelial phenotype when cultured on a two‐dimensional substrate such as plastic or collagen‐coated glass. As part of this dedifferentiation, hepatocytes lose their ability to take up and secrete bile acids. However, culturing between layers of collagen in a three‐dimensional matrix, termed a collagen “sandwich” (Dunn et al. 1989; Liu et al. 1998; Godoy et al. 2013), has been shown to maintain native as well as fluorescent bile acid (FBA) transport, and 3D culturing and FBA uptake has been used to examine mechanisms of cellular transport and drug toxicity (Swift et al. 2010). Expanding on these important studies, we asked whether automated image analysis of hepatocytes in culture could be used to determine the degree to which bile acid transport is maintained under different culture conditions. Rat hepatocytes were isolated and cultured on 96‐well plates in either 2D or 3D collagen matrix configuration and were assayed for their ability to accumulate a series of fluorescent dyes, including the fluorescent bile acid, CDCGamF. Image analysis software was developed to quantify fluorescence intensity of single cells and to eliminate nonviable cells and artifact. These studies took advantage of additional fluorescent dyes, Hoechst (nuclear stain), and Lysotracker (acidophilic vital dye), which were added following a 15 min incubation with fluorescent anions alone. We found that Hoechst, Lysotracker, and other fluorescent probes can interfere with the uptake of CDCGamF, and these were therefore added separately. Collagen overlay can potentially hinder diffusion of solutes. We therefore used a low concentration of collagen (e.g., 0.15 mg/mL) and carefully removed the overlay prior to addition of substrates. The hepatocytes were plated at a density that allows cell to cell contacts and formation of apical domains. Increasing cell density resulted in increased cell death under these conditions (i.e., in the absence of serum and with 0.1 mg/mL of fresh collagen coating). Higher density can be achieved in the presence of serum, although hepatic phenotype and gene expression are reportedly better maintained in the absence of serum in 3D culture (Tuschl et al. 2009).

Seven hours after their isolation, hepatocytes accumulated high levels of fluorescent bile acid (FBA, Fig. 1A and C), and this was not altered by short‐term (4 h) culture between layers of collagen in the 3D configuration (Fig. 1B and D). The *Y* axes in A and B show the average pixel fluorescence intensity of the cytosol of individual cells, with dead or damaged cells excluded (see Methods). The panels (C, D) show representative fields of cells in the FBA channel with the cytosolic ‘region of interest’ outlined. Cells appear round at early time points. Some pixel intensities may appear saturated, but this is due to image scaling. For comparison, we assayed for the accumulation of three other fluorescein‐containing anions: fluorescein (FL), carboxyfluorescein diacetate (CFDA), and carboxyfluorescein succinimidyl ester (CFSE). All these have been shown to be taken up into hepatocytes (Sherman and Fisher 1986; Fujioka et al. 1994; Li et al. 2009), and a quantitative comparison may provide mechanistic insight into the loss of transport activity during dedifferentiation. Accumulation of the base fluorophore, fluorescein, was low for all cases (e.g., 30‐fold lower than FBA fluorescence at 7 h). Although fluorescein can be transported by hepatocytes, it appears to require concentrations in excess of 50 micromolar to give significant signal (Barth and Schwarz 1982). CFDA is nonfluorescent, moderately permeable to cells, and converted into fluorescent carboxyfluorescein by intracellular esterases. It should accumulate in cells with high esterase activity and low transport out of cells (McKay et al. 2002). CFSE, used as a cell tracer, on the other hand, is relatively impermeable to cells but once inside will react with free amines to label cytosolic proteins and be retained. Thus, CFSE will accumulate in cells with high inward transport and should be resistant to export out of cells (Ostrowska et al. 2000).

All fluorescent anions were given at 1 *μ*mol/L and contain the same fluorophore group (fluorescein), yet at 7 h there was 4–5‐fold greater accumulation of FBA than CFDA and CFSE for both 3D and 2D culturing (Fig. 1A and B). This strong labeling of hepatocytes by FBA as compared to the other dyes reflects the presence of bile acid transporters in these cells. By 16 h of culture, FBA accumulation was reduced 5.3‐fold (2D culturing) and 2.6‐fold (3D culturing), indicating that even by 16 h of culture, bile acid transport activity in primary hepatocytes is reduced many fold. After 168 h in 3D culture, FBA accumulation was reduced 3.7‐fold whereas under 2D culture the reduction was 17.7‐fold. Fluorescence of 75 or less was considered too low for robust scoring. Under 2D culturing FBA fluorescence reduced to below 100 by 32 h, whereas under 3D culturing FBA fluorescence was maintained above 300 for the duration of the experiment. Both 2D‐ and 3D‐cultured cells lost their ability to accumulate CFDA at a similar rate. By 60 h CFDA accumulation was very low, although 3D culture showed on average 50% more accumulation for all time points. The loss of CFDA accumulation suggests either that esterase activity is reduced or that export of the fluorophore dominates. In contrast, CFSE accumulation was partially maintained in 3D but not in 2D culture. The change in CFSE accumulation was similar to that of FBA over time in culture in that it decreased from 7 to 60 h but was partially retained through 168 h in 3D culture. We interpret this to indicate that inward transport of both FBA and CFSE is maintained in 3D culture, but that transport of FBA is greater. CFSE is expected to be sequestered, or retained, within hepatocytes by forming covalent bonds with free amines (e.g., lysines), whereas fluorescent bile acids appear be sequestered by binding to cytosolic proteins (Holzinger et al. 1998).

Figure 1 also demonstrates that fluorescent anion accumulation fluctuates through time in culture and appears oscillatory in the figure. This is routinely observed in the laboratory and is not a product of experimental error. For example, FBA accumulation was found to *increase* between 32 h and 48 h of culture for both 2D and 3D conditions despite the overall decreasing trend, and this brief increase was significant (*P* = 0.02 between 3D experiments). Fluctuations through time in culture are also evident in accumulation of CFSE as well as other fluorophores as well as in 2D culturing, although the lower overall signal makes them more difficult to discern. The fluctuations may reflect nutrient levels in the culture media, such as glucose, which can affect nuclear receptor transcription factors such as FxR, HNFalpha and SHP (Godoy et al. 2013). They may also reflect circadian oscillations (Ma et al. 2009), or other heterogeneities in cell expression (Herms et al. 2013) that regulate bile acid transport.

### Nuclear size increases and Hoechst accumulation decreases more significantly in 2D culture

We additionally investigated the relationship between cell shape and ligand accumulation in dedifferentiating hepatocytes. Changes in cell shape are integrally related to metabolic status, cellular electro‐chemical gradients, and transporter activity (Boyer et al. 1992; Hodgkinson et al. 2000). Additionally, thin cells will exhibit lower fluorescence than thick cells at equal intracellular fluorophore concentration. Figure 2A shows that nuclear diameter increased over 168 h of cell culture by 53 and 14% for 2D and 3D culture, respectively. Cell diameter also increased in parallel to nuclear diameter. By 72 h in 2D culture, some cells had migrated into aggregates, whereas other cells had spread very thin, as is frequently observed (Wang et al. 2008). Interestingly, Hoechst 33342, a semipermeable DNA binding cation, is known to be excluded by cells that exhibit the “side population” stem cell phenotype that is seen by flow cytometry. Hoechst excluding cells typically express high levels of exporting pumps, MDR1 (P‐glycoprotein) and ABCG2 (BCRP). In liver, these transporters function as canalicular export pumps (Fausto 2004; Moserle et al. 2010). Figure 2B demonstrates that the average nuclear fluorescence (i.e., the intensity of Hoechst staining) was reduced over time in both 2D and 3D culture, but that this reduction was much greater in 2D culture. To determine whether the reduced intensity was a consequence of thinner nuclei, we measured the total nuclear fluorescence (i.e., integrated pixel intensity of Hoechst stain) and found that it decreased 7.8‐fold by 168 h in 2D culture while it decreased by 1.5‐fold in 3D culture (Fig. 2C). As DNA content should remain constant or possibly increase (De Smet et al. 2001), this indicates that, under 2D culture, the cells became refractory to Hoechst accumulation. This is potentially due to the increase in expression of export (efflux) transporters that occurs during cell culture (Rippin et al. 2001; Zhou et al. 2001; Lundquist et al. 2014).

### Cytotoxic response to hydrophobic bile acids is maintained with 3D culturing

The previous experiments demonstrate that significant accumulation of fluorescent bile acid is maintained under 3D culturing of hepatocytes, whereas under 2D culture, accumulation is reduced to the level of the noise by 32–60 h. To determine whether response to unmodified bile acids is also maintained, we measured the cytotoxic response in dedifferentiating hepatocyte cultures. High levels of bile acids are associated with damage of the liver, and hydrophobic bile acids, such as glycochenodeoxycholic acid (GCDCA), lithocholic acid, and chenodeoxycholic (CDCA) acid, can induce hepatocyte cell death both within the liver and in cultured cells (Hohenester et al. 2010; Woolbright et al. 2014). However, primary hepatocytes rapidly develop resistance to bile acid induced toxicity in culture (Gonzalez et al. 2000; Godoy et al. 2013). We performed bile acid toxicity assays by exposing hepatocytes to the hydrophobic bile acids, GCDCA and CDCA, as well as to the toxic drugs, acetaminophen (APAP) and phalloidin (Phal), for 14 h followed by automated scoring of cell viability (Eguchi et al. 1997). Bile acid and drug concentrations to achieve moderate toxicity were determined from preliminary experiments and from published studies (Hohenester et al. 2010; Chatterjee et al. 2014). Acetaminophen was included as toxicity from this drug is reportedly preserved in 3D‐cultured hepatocytes (Schyschka et al. 2013), although this has been disputed (Farkas and Tannenbaum 2005). Phalloidin was included as an alternate toxin that damages hepatocytes through artificial polymerization of F‐actin leading to disruption of cellular membranes and calcium dependent toxicity (Russo et al. 1982).

Figure 3 demonstrates that the cytotoxic response of hepatocytes to these toxins changes over days in culture and is dependent on whether cells are cultured in a 2D or 3D collagen matrix. On day 0, all toxins showed strong cell death compared to control (although APAP and Ph treatment in 3D culture did not reach significance, *P* = 0.15 and 0.17, respectively), whereas by day 1, hepatocytes in 2D culture had reduced responsiveness to APAP, GCDCA, and phalloidin. By day 2, the 2D‐cultured cells were refractory to all of the toxins. Under 3D culture there was a loss of response to APAP and phalloidin on day 1, but by day 2, hepatocytes regained significant toxic response to all of the agents, although the response appears somewhat diminished. Overall, the studies indicate that 3D culturing increases the level of anion accumulation (Fig. 1) as well as the cytotoxic response to hydrophobic bile acids and to acetaminophen and phalloidin.

### Fluorescent bile acid accumulation is variable from cell to cell and does not correlate with zonal heterogeneity of the liver

**Figure 4.**
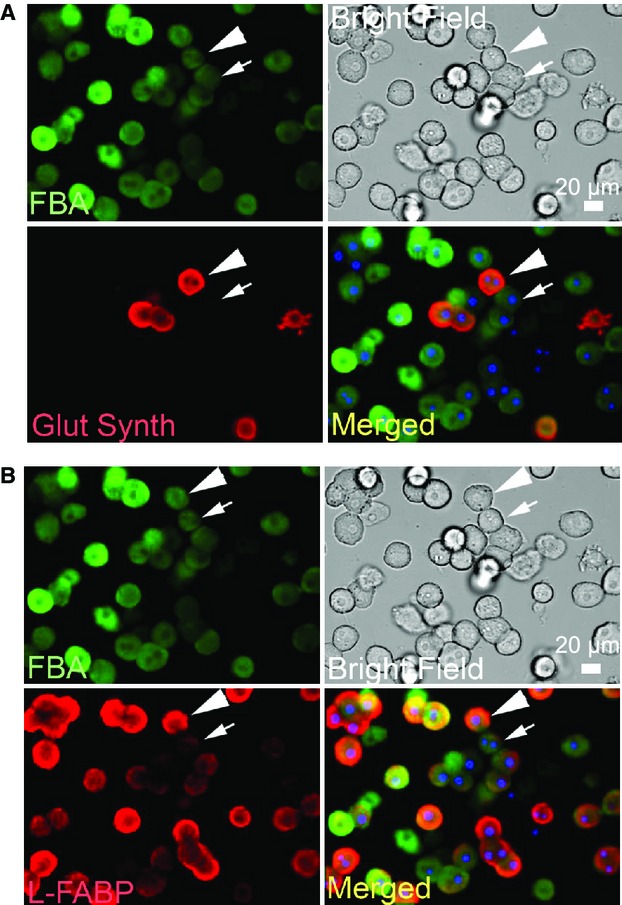
Variability in accumulation of fluorescent bile acid is not strongly dependent on the hepatic acinar zone from which the hepatocyte was derived. Cultured hepatocytes were imaged for the accumulation of fluorescent bile acid and then analyzed for proteins known to localize to either (A), the pericentral (glutamine synthetase, Glut Synth) or (B), periportal (liver fatty acid binding protein, L‐FABP) acinar zones of the liver. Arrow heads and arrows indicate pairs of cells with high and low levels of Glut Synth and L‐FABP protein and approximately equal levels of accumulated FBA. Visual screening and image analysis did not indicate strong correlation of these signals in either negative or positive direction (correlation coefficient −0.03, *n* = 1150 for Glut Synth, and 0.21, *n* = 1150 for L‐FABP).

Several studies have noted that the level of fluorescent bile acid accumulation in hepatocytes varies significantly from cell to cell, and that this is especially apparent in primary cultures (Gebhardt and Jung 1982; Schramm et al. 1993; Milkiewicz et al. 2001; Murray et al. 2011). Understanding this characteristic is important for continued use of this experimental model. The coefficient of variation for FBA accumulation (i.e., the standard deviation divided by the mean, i.e., the typical intensity difference between cells) increased from 13 to 21% from 7 to 168 h under 3D culturing. For Hoechst staining the coefficient of variation for the same cells was 1.7 to 3%. Therefore, FBA has more than sevenfold greater cell to cell variation than Hoechst. Previous studies have indicated that this variation is not due to variable protein levels of the uptake transporters, ntcp and oatp1a1 (Murray et al. 2011). Heterogeneity of the liver is frequently correlated with the flow of blood through zones of the hepatic acinus. To examine for zonation, we performed immunofluorescence correlation experiments using in vitro cultured hepatocytes and antigens known to localize to specific zones. In these experiments hepatocytes were cultured for 4 h, allowed to take up FBA, imaged, then fixed and stained for the localization of glutamine synthetase (Glut Synth, zone 3) or liver fatty acid binding protein (L‐FABP, zone 1). Glutamine synthetase is strictly localized to the region surrounding the central vein in intact liver (Gaasbeek Janzen et al. 1987), and in primary culture only a subset of the cells stained for glutamine synthetase (Fig. 4). However, the results showed that cells expressing high or low glutamine synthetase had equal amounts of FBA accumulation (arrows) and that the intensity of these signals appeared unrelated, with a correlation coefficient near zero (−0.03, *n* = 1150). L‐FABP localizes to the periportal region (zone 1) (Kazantzis and Seelaender 2005), and it can also bind bile acids (Zimmerman et al. 2001) and could serve as an intracellular sequestering agent for fluorescent bile acids. Immunofluorescence correlation experiments showed that although L‐FABP exhibited high and low expression in different cells (Fig. 4B, arrows), only a small positive correlation was observed (correlation coefficient = 0.21, *n* = 1150). These studies therefore imply that variability of FBA accumulation is not strongly related to the zone from which the hepatocytes were derived.

**Figure 5.**
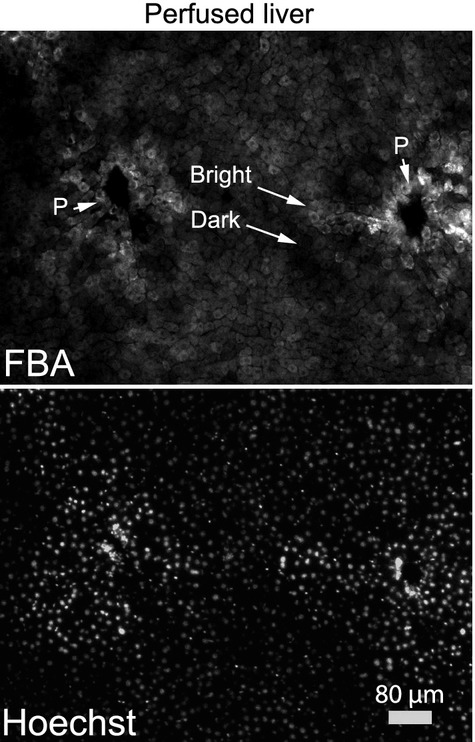
Perfused liver indicates cell to cell variability in fluorescent bile acid accumulation. Accumulation of FBA in the intact liver was visualized by injecting FBA and Hoechst nuclear stain into rat liver portal veins followed by sectioning and imaging the liver. Portal venules are indicated (P), along with examples of cells that retain greater (Bright) and lesser (Dark) amounts of FBA.

To further address cell to cell variability of fluorescent bile acid accumulation in the liver, we performed experiments using frozen liver sections similar to studies by Milkiewicz et al. (Milkiewicz et al. 2001). Livers were injected with FBA followed by Hoechst nuclear stain followed by histological examination. These studies (Fig. 5) showed relatively uneven cell to cell brightness of FBA through the parenchyma with groups of cells retaining more FBA (Bright), and others less (Dark). FBA also concentrated near portal venules (P), presumably because of the higher initial concentration of FBA at the site of entry into the liver, although further exploration may be warranted. Hoechst stained the nuclei of all of the cells in a homogenous manner indicating that the dyes had penetrated the liver. These results are consistent with the previous studies (Sherman and Fisher 1986; Milkiewicz et al. 2001). Imaging of radioactive bile acids has also shown heterogeneous cell to cell accumulation, although a predominant factor was the direction of flow and the dosage of bile acids (Jones et al. 1980; Groothuis et al. 1982).

### Individual hepatocytes with high‐fluorescent bile acid accumulation have high rate of Cell death when subsequently exposed to hydrophobic bile acids

**Figure 6.**
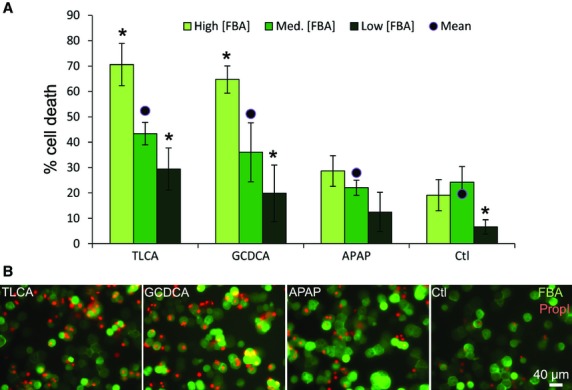
Hepatocytes with high FBA accumulation exhibit high levels of bile acid induced cell death. To correlate the initial FBA accumulation of single cells to their subsequent level of cell death, hepatocytes were exposed to 100 nmol/L FBA, imaged, and then exposed to inducers of cell death, 500 *μ*mol/L taurolithocholic acid (TLCA), 150 *μ*mol/L glycochenodeoxycholic acid (GCDCA), 10 mmol/L acetaminophen (APAP), and then imaged for 30 h. Single‐cell measurements were then sorted by their accumulation of FBA and divided into three groups of equal numbers of cells, high, medium, and low FBA accumulators. (A) The graph indicates that the high accumulators had high cell death when exposed to bile acids but not in control (Ctl). The dot indicates mean cell death of the whole population. (B) Representative images from these experiments showing accumulation of FBA at 0 h, overlaid with propidium iodide (PropI) staining at 30 h, which indicates cell death. *P < 0.05 compared to whole population, student *t*‐test. Bars are standard deviation between image fields.

To examine for consequences of different levels of accumulated fluorescent bile acids within individual hepatocytes, we performed cytoxicity correlation analysis of individual cells using live microscopy. We reasoned that cells with high FBA accumulation should also accumulate high amounts of hydrophobic bile acid, and that the ensuing cellular damage would also be higher in these cells. To accomplish this, primary rat hepatocytes were treated with FBA, propidium iodide, and Hoechst (not shown), imaged, and then treated with bile acids or APAP and imaged over 30 h (Fig. 6). Individual hepatocytes were scored for cell death by an increase in propidium iodide staining using a computer algorithm. For the entire population, the level of cell death was 19.6, 27.9, 52.1, and 52.4% for Control, APAP‐, GCDCA‐, and TLCA‐treated hepatocytes (black dots, Fig. 6), indicating that the treatments induced cell death as compared to control, although cell death response was diminished under these live cell culturing conditions and APAP treatment did not reach statistical significance (*P* = 0.22, 0.002, 0.004 for APAP, GCDCA, TLCA versus control). Cell parameters from each experimental condition were sorted according to initial FBA accumulation and divided into three groups of equal numbers of cells, referred to as high, medium, and low FBA accumulation groups. Figure 6 shows the percentage of cells that underwent cell death in these high, medium, and low groups. In the high FBA accumulating cells, the level of cell death was 19.1, 28.7, 64.7, and 70.6% for control, APAP, GCDCA, TLCA (*P < 0.05 for each compared to the whole population). The high FBA accumulating cells showed a 13.3 and 18% greater cell death than the whole population for GCDCA and TLCA treatments. In control cells, high FBA accumulating cells were not more likely to undergo cell death compared to the whole population (19.1% cell death for high accumulators, 19.6% cell death for total accumulators), indicating that high accumulation of FBA itself did not result in toxicity. Sorting the data another way, it was found that the cells that that underwent cell death had 1.2‐, 1.4‐, 2.2‐, and 1.8‐fold higher initial FBA fluorescence in control, APAP‐, GCDCA‐, and TLCA‐treated cells as compared to cells that survived (*P* < 0.05 for all when comparing FBA mean fluorescence of individual cells that underwent cell death to those that did not within each condition). Interestingly, the low uptake cells for all experiments showed a lower death rate, suggesting that cells with low FBA accumulation may be protected from cell death, a possibility that requires further investigation.

**Figure 7.**
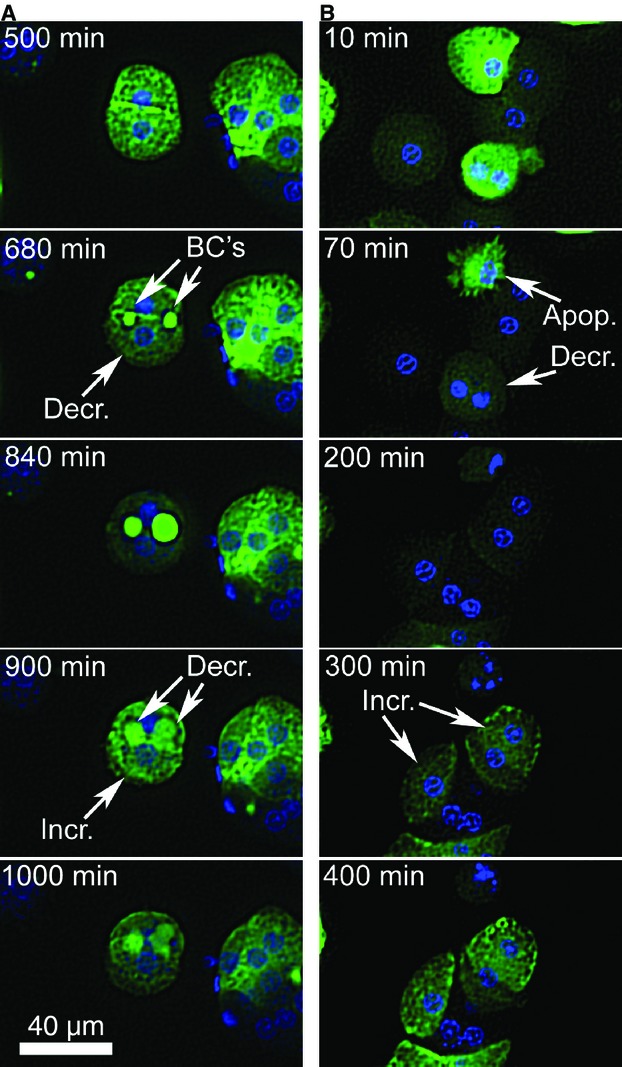
The level of fluorescent bile acid accumulation oscillates independently within individual hepatocytes during primary culture. Example Images from the 30 h experiments of Fig. 6, solvent control, are shown. Spot enhancement filter and contrast adjustment has been applied to the entire frames. (A) An example where individual hepatocytes decrease (Decr.) their FBA fluorescence from 500 to 840 min of observation, accompanied by an increase of fluorescence in bile cananlicular structures (BC's). (B) Examples where individual hepatocytes increase (Incr.) FBA fluorescence at 200 to 400 min of observation. A cell is also seen undergoing apoptosis (Apop.) at 70 min, note the fragmented nucleus.

The studies of Fig. 6 indicate a correlation between high FBA accumulation and high cell death in response to addition of bile acids. We propose that high cell death is due to high accumulation of hydrophobic bile acids. However, we cannot exclude the possibility that FBA accumulates in cells that are sensitive to cell death for other reasons. Indeed FBA can label apoptotic or nonviable hepatocytes (Fig. 7). However, at the beginning of these experiments, all the hepatocytes that were scored were viable as defined by exclusion of propidium iodide and normal intensity and geometry (roundness) of nuclear stain. In addition, the high accumulating cells did not show elevated rates of cell death in control experiments and had only slightly elevated rates of cell death in acetaminophen‐treated experiments, indicating that the high death rate was specific for bile acid treatment. As an additional observation, we found that the level of FBA within individual hepatocytes tended to change over the course of several hours in culture. We found that the addition of bile acids caused an overall decrease in FBA fluorescence over time, potentially related to displacement of FBA by bile acids. In control experiments, however, hepatocytes frequently decreased their cytosol FBA fluorescence, coincident with increased fluorescence in bile canalicular‐like structures (Fig. 7A). They also frequently increased their cytosolic FBA fluorescence (Fig. 7B). These examples indicate that the accumulation of FBA oscillates for individual cells, and that this can be associated with bile canalicular contractions that have been observed in cell cultures and in the intact liver (Gebhardt and Jung 1982; Watanabe et al. 1991; Boyer 1997). These changes in accumulation could relate to cytoskeletal‐based trafficking of uptake transporters, such as oatp1a1 and ntcp, that we and others have shown occurs in hepatocytes, or this may reflect other forms of regulation of bile acid uptake and accumulation (Mukhopadhayay et al. 1997; Sarkar et al. 2006; Wang et al. 2014).

## Discussion

These studies were initiated to further understand the effects of culturing rat hepatocytes between layers of collagen in the sandwich configuration, and to determine whether the fluorescent bile acid, CDCGamF (here termed, FBA), can serve as a marker of hepatocyte phenotype in automated image analysis. In the 3D configuration, the top layer of collagen can form a barrier to solute diffusion. This was overcome by the use of low concentrations of collagen (0.15 mg/mL) and removal of the top layer prior to experimental manipulation. As a technical note, imaging of hepatocytes in the presence of vital dyes and markers of cell death provided an important tool to inspect for cellular damage that can occur during uptake assays. Figure 1 demonstrates that CDCGamF brightly labeled fresh hepatocytes but poorly labeled dedifferentiated hepatocytes. The labeling was maintained though 168 h of culture under collagen (3D). However, even under 3D culture, the intensity of FBA was significantly reduced by 16 h, and it stabilized to levels that were 3–4‐fold less than for 7 h hepatocytes. FBA had much brighter labeling of hepatocytes than the related dyes, fluorescein (30‐fold), CFDA (4.4‐fold), and CFSE (4.8‐fold). It should be noted that other culturing conditions can affect the appearance and cytotoxic response of hepatocytes. For instance, hepatocytes appear to show less spreading when cultured in the presence of serum and on substrates other than collagen (Vinken et al. 2011; Godoy et al. 2013).

At least three levels of variability, or heterogeneity, of fluorescent anion accumulation are observed in these studies; (1) acinar zonal variability, which here did not play a dominant role (Fig. 4); (2) population wide oscillations during the first 72 h of culturing (Fig. 1); and (3) cell to cell variability (Figs. 4,7) and single‐cell oscillations (Fig. 7). In addition to these, liver transporters exhibit significant individual variability between patients (Godoy et al. 2013). Swift et al. (2010) have made efficient use of cuvette‐based fluorescence measurements that avoid single‐cell variability and potential environmental effects on the fluorophore, whereas pioneering image‐based studies of hepatocyte couplets helped provide a basis for understanding transport physiology but tended to avoid analysis of cell to cell variability (Watanabe et al. 1991; Boyer 1997). Here, we demonstrate that automated analysis of populations of hepatocytes exposed to fluorescent anions can be used to generate quantitative data, and that hepatocytes in 3D culture can be analyzed for transport activity for at least 7 days, the first 72 h of which may represent a period of phenotypic adjustment (Figs. 1, 2, 3).

Bile acid and drug‐induced toxicities were maintained in 3D culture and can also be analyzed by automated imaging (Figs. 3,6). This presents an attractive system for measuring the hepatocyte‐specific effects of drugs, as these hepatocytes establish cellular contacts and cell polarity similar to that seen in vivo. Cell to cell variability of FBA accumulation was examined in vitro (Fig. 4) and in vivo (Fig. 5), and this variability increased slightly over time under 3D culturing (e.g., the coefficient of variation increased 13 to 21% from 7 to 168 h in 3D culture). Experimentally, this raises the apparent noise of the system. However, its relevance in liver function is unclear. Here, it was shown that cells with high FBA accumulation experience high rates of cell death when exposed to hydrophobic bile acids. We speculate that the liver may maintain cell to cell heterogeneity of toxin accumulation in order to provide a robust organ wide response to such toxins. Additionally, the regulation of bile acid accumulation by individual cells may reflect the trafficking of uptake transporters to and from the cell surface on actin and microtubule networks (Mukhopadhayay et al. 1997; Sarkar et al. 2006; Wang et al. 2014).

## Conflict of Interest

None declared.
